# How would you like to be helped? A concept mapping study of potential factors promoting integrated approaches for people with mental-health problems to find gainful employment

**DOI:** 10.1371/journal.pone.0357885

**Published:** 2026-10-06

**Authors:** Ard J. van Oosten, Mina Maharadjdin, Roland W. B. Blonk, Alex Burdorf, Bea Tiemens

**Affiliations:** 1 Parnassia Groep, 's-Hertogenbosch, The Netherlands; 2 U-center, Epen, 's-Hertogenbosch, The Netherlands; 3 GGZ Momentum, 's-Hertogenbosch, The Netherlands; 4 Pro Persona Research, Renkum, The Netherlands; 5 Department of Human Resource Studies, Tilburg University, Tilburg, The Netherlands; 6 Optentia Research Focus Area, North-West University Vaal Triangle Campus, Vanderbijlpark, South Africa; 7 Department of Public Health, Erasmus MC, Rotterdam, The Netherlands; 8 Behavioral Science Institute, Radboud University, Nijmegen, The Netherlands; Thammasat University, THAILAND

## Abstract

For enabling people with mental-health problems to find work, an integrated approach is needed. It would combine mental-health services with social services for securing income and social benefits. This study used concept mapping, a mixed-method participatory research methodology that combines qualitative and quantitative techniques to identify factors that would promote such an approach. The participants included (a) experts with lived experience in mental-health problems, (b) client organizations, employers, and the General Employers’ Association Netherlands (AWVN), as well as municipal return-to-work professionals, mental healthcare providers, and the Netherlands Employees Insurance Agency (UWV). Together, they collaborate to guide, support, and facilitate people’s reintegration into the workforce. Participants were recruited between May and September 2018. In Phase One, 63 participants brainstormed how to improve collaboration between the different parties in finding and retaining employment for people with a mental illness. In Phase Two, participants sorted the statements from Phase One into categories based on thematic similarity, and they rated them on priority. Multidimensional scaling and cluster analysis were performed to produce multidimensional maps to visualize the findings. In the brainstorming phase, participants generated 143 statements which were reduced to 60 statements to be included in Phase Two. In the sorting task, six clusters were created as follows: *Provide time and space for collaboration*, *collaborate*, *provide a safe landing for employees*, *focus on common goals*, *facilitate and connect craftsmanship*, and *address the impact of work on health and recovery*. Because experts with lived experience and employers were underrepresented in the development of the concept map, two additional focus groups were organized to verify the results of the concept map. One group included experts with lived experience, and the other one included employers. The results of these focus groups confirmed those of the group concept mapping. Ways to improve an integrated approach are discussed. We distinguished six clusters of potential factors that might foster an integrated approach: “Provide time and space for collaboration”, “collaborate”, “provide a smooth transition for employees”, “focus on common goals”, “facilitate and connect craftsmanship” and “pay attention to the impact of work on health and recovery”.

## Introduction

Having a suitable job affects the health and recovery of people with a mental-health problem. In fact, it plays a central role in the development, expression, maintenance of and recovery from a mental health problem [1–5]. On the one hand, poor working conditions, including discrimination and inequality, an excessive workload, a lack of control and job insecurity pose a risk to employees’ mental health. On the other hand, having satisfying work can promote connections to the broader social and economic environment, enhance well-being, and provide a source for individuals’ satisfaction and sense of accomplishment.

Many workers struggle with mental health problems. An estimated 15% of working-age adults have a mental disorder at any point in their lives [6]. Depression and anxiety are estimated to cost the global economy a trillion U.S. dollars each year, which is predominantly driven by lost productivity. People suffering from severe mental illness are largely excluded from the workforce, even though being gainfully employed is important for their recovery.

In 2015 The Netherlands opted for a decentralization of social policy from central government to municipalities to increase policy efficiency and to reduce costs [7]. The consequences of decentralizations were expected in different areas. First, it was expected that by concentrating implementation tasks in the social domain, municipalities would be able to match the supply of services to the demand in a more targeted way. Second, incentives for efficient implementation would become stronger because municipalities would bear the risks associated with noncompliance. Previous experiences revealed that shifting to risk-bearing implementation by municipalities might lead to a decrease in the volume and/or price of the use of facilities.

Decentralization has occurred in many areas. In the social domain, the objective was to achieve a better integration of care for adults with mental health problems, who were the target group of this study [8]. Greater cooperation between different municipality policy domains, such as work, benefits and care, was expected to lead to a better provision of services for citizens. This appears obvious because people with mental health problems who depend on benefits or support from the municipality often have multiple problems in different life domains, such as physical and mental health care, child rearing, housing, and income. Municipalities, therefore, have to deal with many different professionals from different organizations, and this requires an integrated approach. However, despite the policy measures that were introduced, the development of integrated care with a focus on participation in work by people with mental-health problems is still lagging.

There is no single definition of integrated care. Keywords used in the international literature that may refer to an integrated approach include “cooperation”, “common goal”, “the result of integral cooperation”, “vertical and horizontal integrality”, and “gradations of integral service delivery” [9]. Vertical integration refers to the cooperation between different actors within one domain or sector. This includes, for example, cooperation within different services in youth care, ranging from prevention and basic support to specialized treatment, residential care, and aftercare. This ensures that young people will receive continuous, coordinated help throughout their care journey, thereby improving outcomes and avoiding gaps in support. Horizontal integration refers to the bringing together of separate services, professionals and organizations from different domains or sectors. This can relate, for example, to cooperation between work and income and debt assistance. Integrated care is seen as an umbrella term for various principles and ideas that seek to better coordinate care around people’s needs [10–12]. Still, however, more insight is needed to explore what an integrated approach might entail.

In this paper, we examine how cooperation between professionals from different domains can be further improved to develop effective integrated interventions that promote employment opportunities for people with mental health problems. Specifically, we aim to identify the factors that potentially promote an integrated approach across the domains of income, benefits, and care to support adults with mental health problems in obtaining and maintaining paid employment. Adults with mental health problems experience a wide range of diagnosable conditions that affect their thinking, emotions, mood, and behaviour, which may influence their ability to function in daily life, including work, relationships, and social interactions. By identifying these facilitating factors, this study seeks to contribute to the development of effective integrated interventions that enhance employment participation for this population.

## Methods

In this study, we used concept mapping to develop a conceptual framework. Concept mapping [13–15] is a structured method for collecting, organizing, and visualizing different participants’ ideas regarding a particular theme. These components are combined to ultimately arrive at a unifying conceptual framework. In the present study, we focused on a conceptual framework that would facilitate (a) an integrated approach by all professionals from mental and physical health care, and (b) participation in work that would support people with mental-health disorders. The concept map combines a quantitative with a qualitative approach to data collection and analysis.

To reflect the diversity of perspectives and to provide a reliable concept map, 20–30 participants were required to prioritize and sort ideas into categories [14].

### Project structure

At the start of the project, a research team, steering committee and a taskforce were organized. The research team comprised three project members (AvO, MM, BT). The steering committee included board-certified members from the various stakeholder organizations. The task force consisted of 11 members recruited from the members at the executive and middle-management levels to coordinate the actions needed to conduct the study.

### Data collection

Data were collected using Concept System Global MAX^TM^, a secure web-based platform for group concept mapping. Online consent was obtained after participants were informed of the details of the study. Participants were able to withdraw their responses until they clicked the “save and submit” button. All responses were anonymous and, therefore, not retrievable after submission. The project was executed in Dutch.

### Study phases

The study consisted of seven phases: (1) Preparation, (2) Invitation to Participants, (3) Statement Generation (4) Clustering and Prioritizing, (5) Quantitative Data Analysis, (6) Interpretation of the Results, and (7) Focus-Group Discussions to enrich interpretation of the findings.

#### Preparation.

The research team defined the purpose of the study, identified the target population, and formulated the questions to be addressed.

### Inclusion and exclusion criteria

Participants were eligible if they were professionals or experts by experience who were actively involved in daily practice and had demonstrable expertise in the domains of income, benefits and care. Participants were selected to ensure representation of the different domains involved in integrated employment support for people with mental health problems. Individuals who were not involved in daily practice or who did not have relevant expertise in the field were not eligible to participate.

#### Invitation to participants.

The steering committee and task force identified and nominated eligible participants from the different stakeholder domains involved in employment support for people with mental health problems. In total, 106 participants were invited to take part in the concept mapping.

Participants were invited from among experts with lived experience, client organizations, employers, the General Employers’ Association Netherlands (AWVN), professionals from municipalities who guide clients in returning to work, mental healthcare providers, the Netherlands Employees Insurance Agency (a governmental agency responsible for administering employee insurance schemes and executing labor laws in the Netherlands).

Potential participants were approached by telephone to participate in the online concept mapping. After their approval, the participants were formally invited by email, and after two weeks had elapsed they were reminded of their agreement to participate in the concept mapping. Recruitment occurred between May and September 2018.

### Qualitative data analysis

#### Generating statements.

The qualitative data were collected during the concept mapping sessions, in which participants generated statements in response to the focus prompt.

After the statements had been generated, the research team undertook a structured and iterative synthesis of them, using the procedure described by Kane and Trochim [16].

Duplicate statements and statements that did not answer the research question were eliminated. Statements with the same meaning were merged by consensus.

As stressed in the literature [15], editing of the statements was minimized, and an attempt was made to maintain each statement’s original meaning, while ensuring that no ideas or thoughts were removed during this process.

#### Clustering and prioritizing.

In Phase Four, a clustering activity was conducted in which each participant independently grouped the statements into what they perceived to be similarity in themes. Next, the participants provided a label for each group of statements. In the final step, the participants were asked to prioritize the statements on an eight-point Likert scale ranging from *completely unimportant* (score of 0) to completely important (score of 7).

The focus group discussions were analyzed using an inductive qualitative content analysis. Two researchers independently reviewed the transcripts, identified key themes related to the interpretation and practical implications of the concept map, and discussed discrepancies until consensus was reached. The qualitative findings were used to enrich and contextualize the concept mapping results and are presented alongside the concept map to provide a more comprehensive understanding of the identified factors.

#### Quantitative data analysis.

After the statements had been sorted and rated, they were converted into quantitative data using multidimensional scaling analysis to construct a point map [15]. The horizontal and vertical axes of the point map depict the groups of statements, which were distributed in a two-dimensional space. Each point represents a statement or an outcome. Areas where multiple points are close together indicate that the participants were more likely to agree that these statements shared a common theme. A cluster analysis of the point map, which was examined via cluster bridging values along with the cluster map and the research team’s judgements, was used to identify the final number of clusters [14].

The stress value was used to assess the goodness of fit of the map that resulted. The stress value is a numerical indicator of how well a multidimensional scaling map represents relationships, or similarities, among a set of items.

The data were analyzed using Concept Systems Global MAX^TM^ software.

#### Interpretation of the results.

The (x,y) points on the map were used in a hierarchical cluster analysis to determine how well the statements were grouped together in terms of their similarity.

A procedure for determining the number of clusters was used to interpret the concept map. However, no exact criteria are available for selecting the final number of clusters [14]. The procedure began with a ten-cluster solution and successively increased or decreased the number of clusters by one. After each step, the different solutions were examined for their face validity. A decision was made about whether in each solution the merger of clusters appeared to adequately represent the original data that the participants provided and whether it formed a coherent system of clusters. This procedure was continued until an illogical merger of two clusters resulted, i.e., the statements did not share a common meaning.

#### Focus groups to enrich the findings.

Experts with lived experience and employers were underrepresented in the group of participants; therefore, after the concept map had been completed, we organized three focus groups to resolve any discrepancies. Two of the groups comprised experts with lived experience, and one of them included employers.

In February and March, 2020 brainstorming occurred in two of the groups with seven and eight experts, respectively, with lived experience. They were asked to describe the current level of cooperation among the various parties involved and then to depict the ideal situation in which there was an integrated approach. The following three questions were further discussed: Which organizations have been involved with you in identifying or retaining employment for individuals with mental-health problems? What things function well in your work with these organizations? What can be improved in the collaboration?

In February 2020, we brainstormed in one group with eight employers about these two questions: What would help you most to retain employees with mental health problems in employment? What might help you to find work for people with mental health problems?

Reports were then made of the focus-group discussions. They were distributed to the members of the focus groups for them to check for accuracy.

### Ethical review

This exploratory, mixed-method study did not require formal ethical review by an institutional review board because the study did not involve medical-scientific research. The participants were not subjected to a medical intervention, nor were they required to comply with behavioral regulations. Prior to participation, each participant provided informed consent.

## Results

### Preparation

The researchers formulated the following research question: How can cooperation among the parties involved in finding and retaining work for people experiencing mental health problems be improved? To answer the question, the participants were given the following explanation: “Many different professionals and organizations are involved in retaining and finding work for people with a psychological vulnerability. To support these professionals and organizations, cooperation is necessary among the following: the municipality, the UWV, employment agencies, employers, and healthcare providers, such as the patient’s general practitioner, psychologist, psychiatrist or nurse.”

Statements were then generated by asking the participants to complete this sentence: “The cooperation among these parties in finding and keeping work for people with a psychological vulnerability can be improved by....”

### Participants

A total of 106 people were initially invited to participate in the study. Sixty-three participants responded and submitted as many statements as they could. Forty-two of these participants answered the general questions about their role, sex and years of work experience. Details about participants’ demographic characteristics are shown in Table 1.

**Table 1:** Characteristics of 42 participants involved in the concept mapping.

Characteristics	Participants
Role	n (%)
Experts with lived experience	5 (12)
Employers	2 (5)
Professionals from the municipality	9 (21)
Professionals from mental-health services	9 (21)
Professionals at Netherlands Employee Insurance Agency	10 (24)
Others	7 (17)
Sex* n (%)	
Male	16 (38)
Female	27 (62)
Years of work experience	
Average	10
Minimum	1
Maximum	37
Median	8

* Sex (male or female) was designated as a dichotomous variable, but currently we designate gender more broadly.

### Generating statements

The 63 participants generated 143 statements which in their opinion promoted an integrated approach in the domains of income, benefits and care. The original statements were merged into a subset of 60 statements. Variability in the data was ensured by the number of statements generated and because a wide range of perspectives had been identified.

### Clustering and prioritizing

A total of 28 of the 63 participants completed the response sorting task, and 27 of them prioritized the statements. Thirteen of the participants had direct client contact, and 14 of them were managers.

Translation of the statements were carried out by the first author, and the other authors compared the translations with the original text.

### Quantitative data analysis

The MDS analysis of the sorted data generated the point-map on which all further analyses were based (Fig. 1). A final multidimensional scaling stress value of 0.2472 was obtained. This value, which falls in the range of from 0.205 to 0.365, is considered typical and acceptable for concept mapping studies. The 27 participants who were involved in the prioritizing and sorting of the tasks was sufficient to reflect the diversity of perspectives and to provide a reliable map [14].

**Fig 1:**
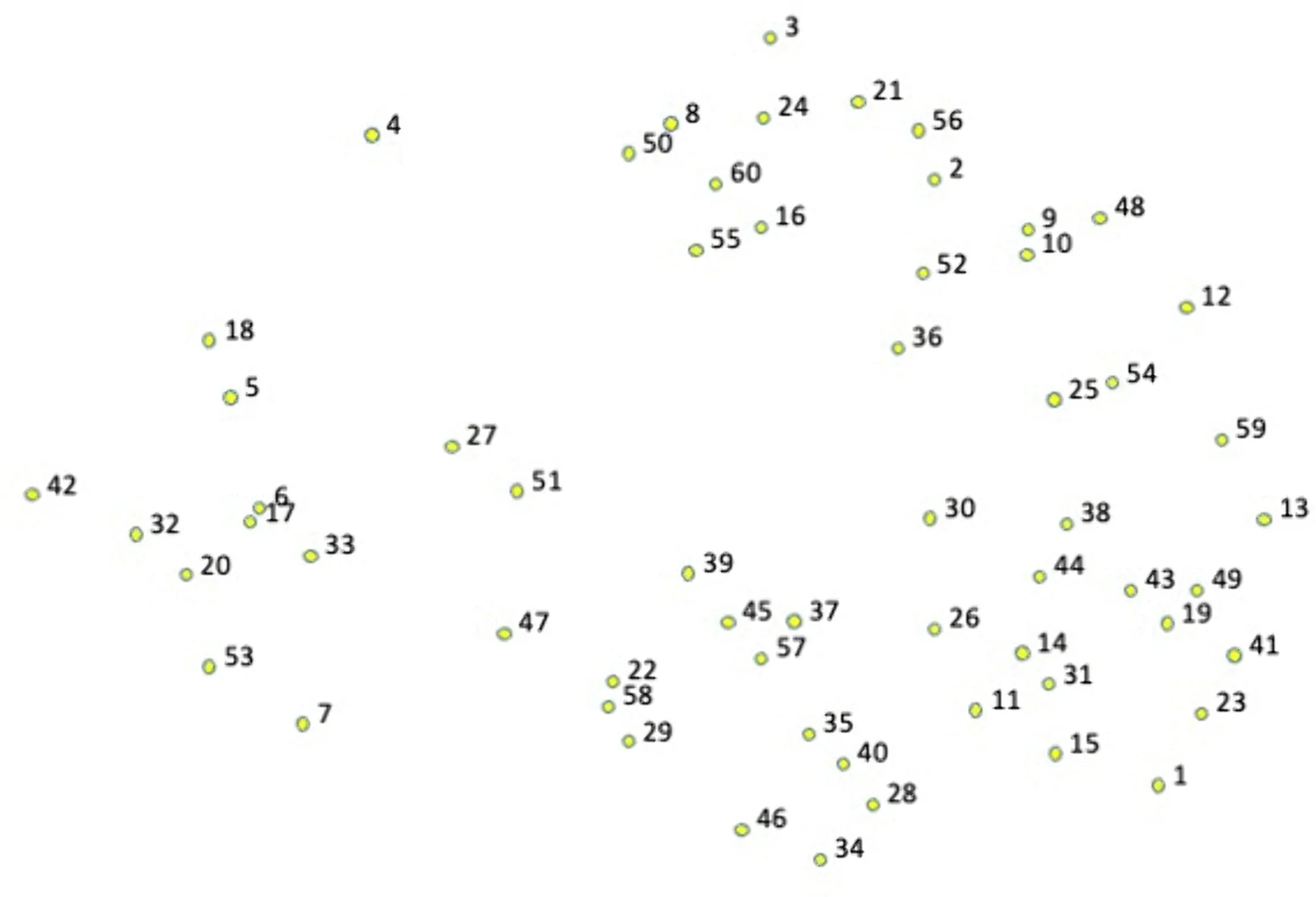
Point map. The 60 statements are displayed at (x,y) points.

### Interpretation of the results

The map displays 60 of the statements. A six-cluster structure was judged to be the best fit for the results (Fig 2). The names of the clusters were determined on the basis of a consensus in consultation with the task force and steering group and were based on the statements included in the clusters.

**Fig 2:**
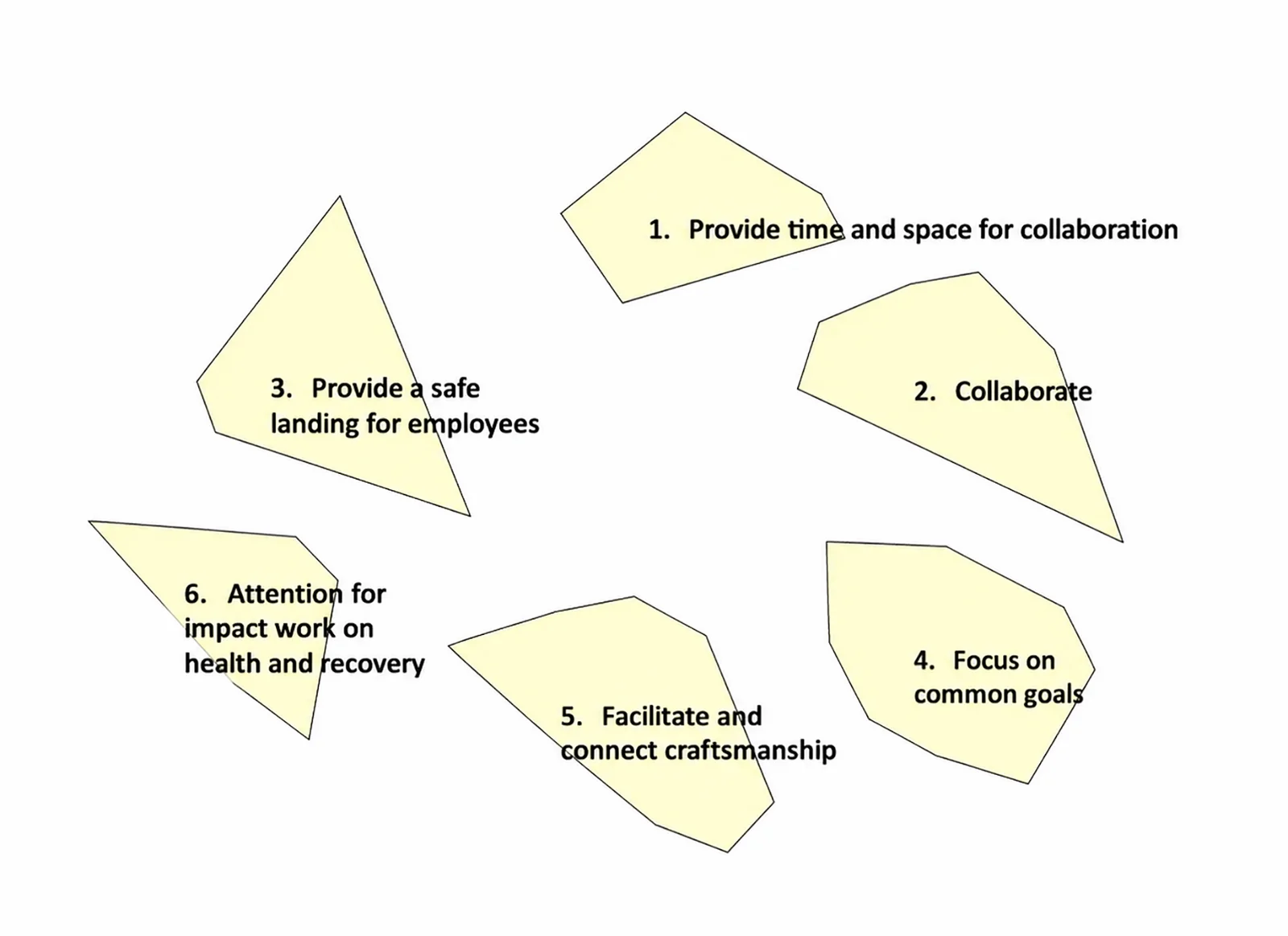
Cluster map. This figure shows the six clusters of statements. Smaller clusters depict a more coherent concept, whereas larger clusters are relatively more heterogeneous.

The multidimensional maps generated by the software are the visual representation of the data and the results of the project. They indicate how the participants perceived the statements in terms of their similarity and importance.

Based on the content of the statements, the six clusters were assigned the following labels and descriptions:

Cluster 1. Provide time and space for collaboration. Invest in collaboration by providing time and space to focus on the client’s needs, specific situation and mental capacity.

Cluster 2. Collaborate. Structure the organizations involved around clients’ need, situation and mental capacity.

Cluster 3. Provide a smooth transitions for employees. Make employees feel welcome. Personal contact with employers can help to lower the threshold for employees with mental illness, so that the client functions well in the organization.

Cluster 4. Focus on common goals. Work with a single plan for recovery in which the actions to be taken within the various domains are specified. Define roles, tasks and the expertise required and decide which partners are needed for the guidance and treatment of the client.

Cluster 5. Facilitate and connect craftsmanship. Connecting craftsmanship refers to collaborating closely with the employees who interact directly with clients in the workplace.

Facilitate and connect craftmanship when developing a policy and ensure that clients, professionals, managers and experts with lived experience are involved. Through frequent case consultations in which different disciplines review cases, the approach that is required will become apparent.

Cluster 6. Pay attention to the impact of work on patients’ health and recovery. Educating all parties helps them to understand the importance of working together and sharing results. For employers, it means understanding what organizations can offer people with a mental illness and the benefits of doing so. For mental health providers, it means understanding the importance of discussing employment within patients’ treatment and how this contributes to patients’ health and recovery. For municipalities and UWV, it is about how the organizations that are involved can be better organized around clients’ needs, situation and mental capability.

### Prioritizing

Twenty-seven of the 63 participants rated all 60 statements. The five statements ranked highest on Importance were “Listen carefully to the candidate and ask questions and not improvise for the person” (mean rating of 6.30); “Don’t restrict executives because of bureaucratic barriers” (6.30); “Think in positive terms like *opportunities*, *trying out* and *learning* instead of in negative terms like *limitations* and *failures*” (6.11); “Explore with the client what the client is capable of doing and what the person’s limitations are” (6.08); and “By collaborating so that the client receives unambiguous information and advice” (6.07).

Statements in the first cluster received the highest ratings on Importance, whereas statements in the sixth cluster received the lowest ratings on Importance. On Importance, the highest rated clusters were: “Provide time and space for collaboration” (mean rating of 5.80); “Collaborate” (mean rating of 5.72); “Provide a safe environment for employees” (mean rating of 5.65); “Focus on common goals” (mean rating of 5.58); “Facilitate and connect craftsmanship” (mean rating of 5.17); and “Pay attention to the impact of work on clients’ health and recovery” (mean rating of 5.15).

Table 2 presents the different clusters with their mean ratings and corresponding items. Participants’ scores on Predictive Value are also included.

**Table 2:** Clusters, mean ratings, and corresponding statements.

Cluster (mean score of expected predictive value as scored by the participants)	Number and content statement	Participants’ mean ratings
Provide time and space for collaboration (5.80)	24. Listen carefully to the candidate and ask questions and do not improvise for the person. 55. Think in positive terms like *possibilities*, *trying out* and *learning* instead of in negative terms like *limitations* and *failures*. 8. Explore with the candidate what the candidate can do and what the candidate’s limitations are. 21. Focus on the needs, specific situation and mental capability of the candidate. 2. Seriously consider employees’ and job seekers’ wishes regarding employment. 16. Inform the candidate in advance of the financial consequences of finding work. 50. Identify the work capability of the person with a mental vulnerability. 60. Inform the candidate in advance about the various parties who can help and who is responsible for what. 56. Involve the candidate in the process, for example, by inviting him or her to attend consultations with other parties. 3. Put the candidate back in the driver’s seat. For example, the candidate could himself or herself schedule appointments using a digital system.	6.30 6.11 6.08 6.04 6.00 5.81 5.63 5.59 5.41 5.07
Collaborate (5.72)	54. Coordinate the actions of one another so that the candidate receives unambiguous information and advice. 52. Have a permanent point of contact for the candidate. 48. Regard the candidate as the starting point and organize the other parties around him or her. 36. Deal seriously with signals from both candidates and employers/supervisors when they encounter difficulties. 25. Oversee the candidate’s consent to information sharing. 59. Keep the focus on the common goal in every consultation. 12. Provide customized services, which might mean sacrificing speed or quantity. 9. Do not make hasty decisions based on the latest information about the candidate. 10. Involve the candidate’s social network. 13. Consider the judgment of professionals.	6.07 5.96 5.85 5.81 5.74 5.67 5.59 5.59 5.48 5.44
Provide a safe landing for employees (5.65)	5. Maintain personal contact with employers to lower the threshold for employing people with mental vulnerabilities. 4. Show how the candidate can add real value to the organization’s interests. 18. Through work adaptations, help employers to create workplaces/jobs for the target group. 27. Before the candidate begins, conduct a workplace analysis in which necessary requirements and possible concessions are discussed with various stakeholders (e.g., supervisor, colleagues) 51. Facilitate employers’ endeavors in the best possible way.	5.85 5.76 5.74 5.56 5.37
Focus on common goals (5.58)	23. Executive workers should not be constricted by bureaucratic barriers. 15. Subordinate organizations to the common goal of finding and retaining employment by creating flexibility to achieve it (e.g., funding, rules, etc. are adjusted accordingly). 19. Actively involve reintegration agencies to facilitate the candidate’s goals. 1. Solicit management’s support for professionals in shaping the collaboration. 49. Meet in person to learn about each other’s point of view; to gain respect for each other’s expertise; and to keep lines of communication open. 26. Ensure a positive atmosphere between the collaborating organizations in which appropriate feedback can be given and received. 43. Be aware of which partners are active in guiding and treating the candidate. 44. Be accessible to one another. 30. Work with one recovery plan in which the actions in the different domains and the persons involved are specified. 31. Organize the chain of command considering everyone’s interests. 38. Checking with one another about whether the goals and expectations are accurate. 41. Have a shared vision in which roles, tasks, and required expertise are specified. 14. Give the party with the most relevant knowledge the role of case manager. 11. Appoint a regional interviewer who assesses the case and assigns it to the appropriate party.	6.30 5.96 5.81 5.78 5.78 5.78 5.78 5.77 5.59 5.52 5.48 5.51 5.31 4.37
Facilitate and connect craftsmanship (5.17)	45. Involve professionals, managers, clients, and experts with lived experience in the policymaking. 28. Ensure there are sufficient financial resources for the parties to work together effectively. 57. Match supply and demand in the workloads. 58. Have information easily accessible to all parties (e.g., web-based). 37. Schedule frequent case meetings in which the different disciplines are actively involved. 34. Coordinate different funding streams (e.g., WMO, Health Insurance and reintegration funds) and then jointly purchase IPS. 40. Support organizations working with IPS. 39. Be informed about the laws and regulations and share this this information with all parties involved. 22. Exchange information with one another on, for example, implementation of the General Data Protection Regulation (AVG), practical matters, and the nature of the service. 47. Utilize IPS and the Wellness Recovery Action Plan (WRAP). 46. Have a national approach instead of a municipal one. 35. Have a collaborative arrangement in which, with the consent of the candidate, the core problem is discussed. 29. Have an alternative arrangement for sick calls in case the candidate relapses, whereby the job coach and fact team have a role to play in the candidate’s recovery, and the person is not treated as a regular employee would be.	6.00 5.70 5.63 5.54 5.37 5.30 5.19 5.15 5.12 4.85 4.65 4.48 4.26
Attention to impact of work on health and recovery (5.15)	20. Increase awareness of Individual Placement and Support (IPS) among the parties involved. 32. Educate all parties about not viewing mental vulnerabilities as taboo. 42. Train employers, especially the direct supervisor, how to function in the target group. 17. Make employers better known and in touch with the target group by inviting them to be a part of the GGZ organization, providing them with information, and involving candidates (who are already working or looking for work) in the process. 53. Encourage/utilize the support of Social Return on Investment (SROI) for this target group. 33. Using a good business case, make the benefits of IPS clear to all stakeholders. 7. Offer special Vocational Guided Learning pathways that involve fewer hours. 6. Tailor the GGZ organization’s website for employers, so that it is clearer what the company can do and what the benefits of this would be.	5.59 5.48 5.31 5.26 5.22 5.11 4.67 4.56

### Focus groups for enriching the findings

#### Experts with lived experience.

Experts with lived experience depicted the current collaboration between the various parties involved and then formulated the desired situation that included an integrated approach. An example or this is shown in Fig 3.

**Fig 3:**
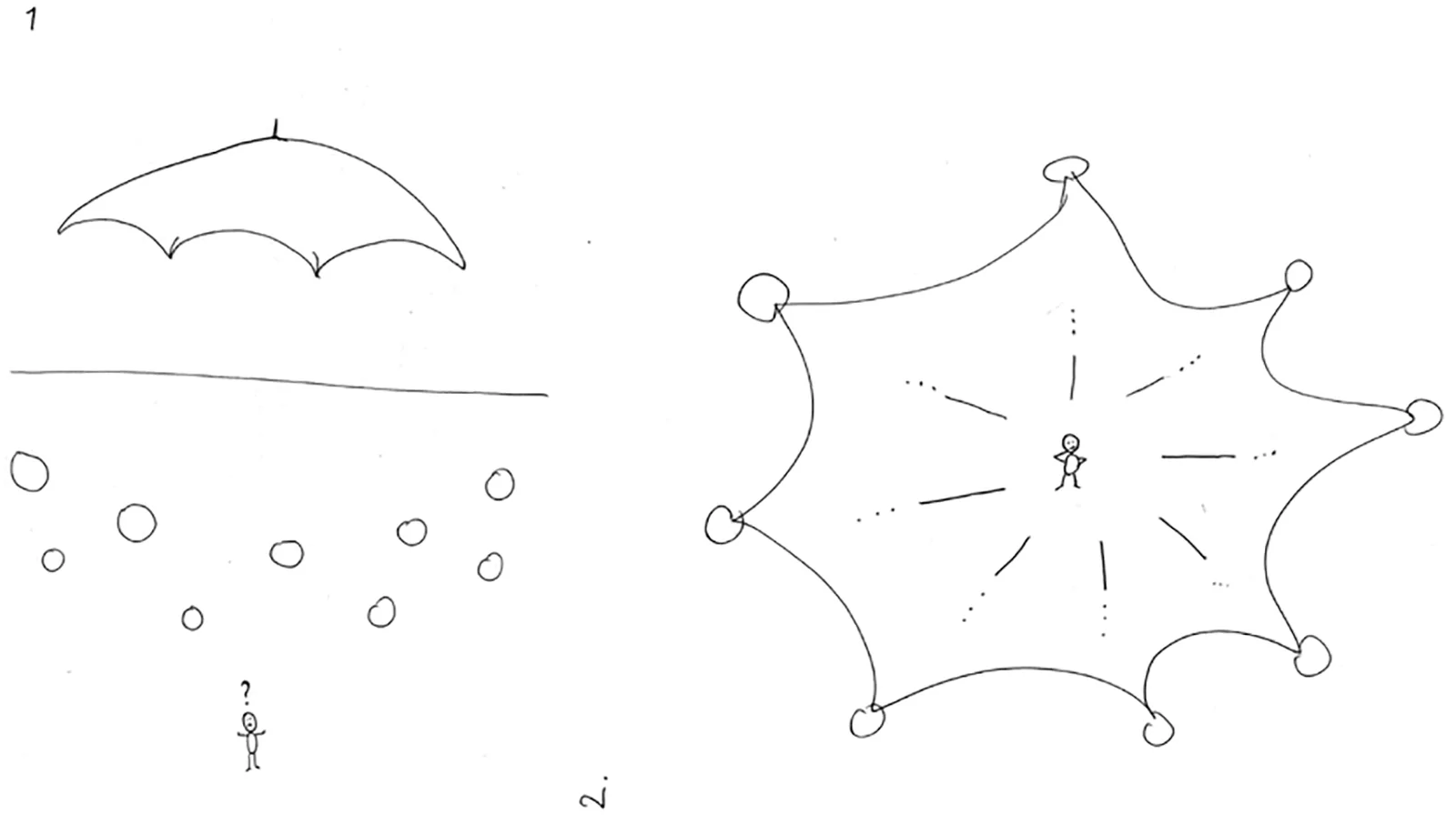
Depiction of the client’s current and desired situation.

The illustration in Fig 3 indicates that in their current situation, the parties involved are not connected to one another and the client does not know what to do. It does appear that the client is involved and able to avoid difficult situations whenever possible. In the ideal situation, the client would be surrounded by and supported by all of the parties involved.

For achieving an integrated approach, the experts by experience emphasized the importance of allocating time and space for cooperation while using a positive approach and a person-centered attitude. The experts by experience suggested, for example: “Have a trustful starting point and don’t start with doubts about others’ sincerity.” “To build trust, the first step is to connect with our needs and to explore together how our work can be valued.” “Consider possibilities instead of impossibilities.” “Make use of experts with lived experience to better meet clients’ needs.” “Consider before taking any action whether the action would make the client stronger. How could this client be helped?” “Provide tailored care that matches the person’s needs and does not stop when employment has been secured.”

The outcome of the clients’ focus group was consistent with the results shown in the concept map. It was especially consistent with Cluster 1 (“Provide time and space for collaboration”) and with Cluster 2 (Collaborate).

#### Employers.

To provide employees with a smooth transition, employers indicated that an integrated approach would be optimal. It would provide a positive mindset and allocate support as exemplified by the statement, “How can we help you?”

Adequate preparation would also be needed. Employers prefer to have a clear profile of the candidate in advance, and they would be asking questions such as: What value can this employee bring to the organization? What does this person have to offer and what would be needed for the candidate to function well? How many hours per week could the person work, and what would be the maximum time required for the person to travel to work? Who would be involved in making the decision? Would there be any kind of financial compensation? Employers want to minimize the possibility of surprises. Good preparation would also mean that job coaches are in place; workplace adjustments have been made where necessary; it would be clear who could support the employer; and expectations would be well managed.

To be able to understand the impact of work on clients’ health and their recovery, employers would want to know more about the role of the employer in an integrated approach. This would include knowing where the employer’s responsibilities would start and end, and when other professionals should be consulted.

Employers indicated the need for a tool that would provide them with guidance when counseling people with a mental illness. For example, what should be discussed? How would agreements be made, and when would they be executed? What questions should be asked, such as what would and would not be possible and how would such issues be dealt with?

Training would also be required. Employes mentioned themes such as: Getting to know yourself better as a supervisor, including identifying your biases. How can you increase your knowledge about mental health problems? How can you best stay in touch with your employee and with caregivers/coaches and their families? To what extent should the employer be involved in the treatment or be informed about it? How much influence does the employer have on the treatment? Or should the employer not be involved in such issues? What could be done if the employee is not productive? What signals matter? For instance, how can I know that an illness is just the common flu and nothing more? What questions can be asked to get greater clarity about the underlying problem?

The employer would like to be able to refer the employee to other parties when problems arise. For example, what should be done if an employee relapses?

Finally, employers mentioned that it would be important for their efforts to be worthwhile. These include providing time, space, and money to invest in employees with a mental health problem.

The suggestions arising from the employer focus group align well with Cluster 3, “Provide a smooth transition for employees” and Cluster 6, “Pay attention to the impact of work on health and recovery”.

## Discussion

In this study, we examined how cooperation between professionals from different disciplines might be improved. We did so by asking stakeholders what would be needed to develop an integrated approach to foster the participation of adult clients with mental health problems in the labor force. We aimed to understand the underlying factors that might promote these individuals’ employability. Group concept mapping was used to achieve our objective.

From the concept map, we distinguished six clusters of potential factors that might foster an integrated approach: “Provide time and space for collaboration”, “collaborate”, “provide a smooth transition for employees”, “focus on common goals”, “facilitate and connect craftsmanship” and “pay attention to the impact of work on health and recovery”.

Each cluster identified several factors that might foster an integrated approach.

### Provide time and space for collaboration

Participants mentioned that when supporting someone in finding or keeping a job, especially someone with mental health problems, it is essential to listen attentively to each person’s views: “Listen carefully to the candidate and ask questions and do not improvise for the person.” Only by really listening can you understand what is happening and what matters to the person. We see a connection here to the concept of *active listening*, a cornerstone of Carl Rogers’ client-centered therapy [17]. Active listening involves providing a nonjudgmental, empathetic environment that fosters personal growth and self-understanding.

The following statements from this cluster exemplify this point of view: “Think in positive terms such as *possibilities*, *trying out* and *learning* and instead of in negative terms like *limitations* and *failures*.” “Explore with the candidate what the person can do and what his or her limitations are.”

We recognize the need for a strength-oriented and solution-oriented approach [18]. This approach focuses on individuals’ strengths and resources, promoting self-efficacy and resilience by emphasizing solutions and positive outcomes rather than problems. It is also related to self-efficacy, a psychological concept introduced by Albert Bandura which refers to people’s belief in their own ability to succeed in specific situations or to accomplish a particular task [19].

Statements such as: “Focus on the needs, specific situation and mental capabilities of the candidate” and “Take the wishes regarding employment of both employees and job seekers seriously” are related to the concept of person-centered and individualized support [20]. The person-centered and individualized support emphasizes the need to reorient health systems according to individuals’ needs, thereby promoting equity, quality, and responsiveness in the delivery of care. It is consistent with the view that the World Health Organization (WHO) has been strongly advocating: A renewed focus on service delivery through an integrated and people-centered lens is critical for achieving this, particularly for reaching underserved and marginalized populations to ensure that no one is left behind [21].

The statements: “Inform the candidate in advance of the financial consequences of finding work” and “Inform the candidate in advance about the various parties that can do something for him or her and who is responsible for what” are in line with the concept of transparency and informed decision-making [22]. Providing clear, accessible information enables individuals to make informed decisions about their care and their employment. This fosters autonomy and empowerment.

Two of the statements are related to clients’ empowerment and self-determination [23]: “Make the candidate a participant in the process by, for example, inviting the person to attend consultations with other parties” and “Put the candidate back into the driver’s seat. For example, allow candidates to schedule appointments themselves using a digital system.” Such actions would enable individuals to take control of their recovery and employment journey. This, in turn, would enhance the person’s motivation and engagement and lead to a better outcome.

### Collaborate

Regarding collaboration, participants were strongly in favor of a person-centered approach: “Take the candidate as the starting point and organize involved parties around him or her.”

Participants’ informed consent is a vital element in the collaboration [24]: “Solicit the candidate’s consent to information sharing.”

Professionals must secure the candidate’s informed consent before sharing any information, thereby respecting the person’s privacy and autonomy. In addition, decisions should be based on the best available evidence [25] and combined with clinical expertise and patient values: “Include the judgment of professionals.”

### Provide a smooth transition for employees

Participants indicated the following as requirements for arriving at the workplace in an appropriate manner: “Enable personal contact with employers to lower the threshold for employing people with a mental vulnerability.” It is essential to establish personal contact with employers, thereby reducing perceived barriers and building trust.

“Show that the candidate can add real value to the organization” indicates the candidate’s potential to contribute in meaningful ways to the organization’s goals and helps shift the focus to the person’s capabilities rather than his or her limitations.

“Before the candidate begins, conduct a workplace in which necessary requirements and possible concessions are discussed with various stakeholders (supervisor, colleagues).” Conducting a thorough workplace analysis ensures a clear understanding of the job requirements, and it identifies necessary modifications.

The statements “Work adaptations help employers create workplaces/jobs for this target group” and “Facilitate employers in the best possible way” show the importance of

supporting employers with tailored work adaptations, which would further enable the creation of inclusive roles suited to this target group. Ultimately, providing comprehensive facilitation helps employers feel confident and capable about offering these individuals sustainable employment opportunities. Several elements of the concept of *individual placement and support* (IPS) are reflected in this description. IPS directly addresses the importance of integration of health care and employment services and cooperation between different specialists by integrating employment specialists into health teams [26,27].

### Focus on common goals

Participants suggested a focus on finding and retaining sustainable employment goals: “Subordinate the organization to the common goal (finding and retaining sustainable employment), thereby creating more flexibility for achieving it (i.e., funding, rules, etc. would be adjusted accordingly). Seek freedom from bureaucratic barriers: “Workers should not be limited by bureaucratic barriers,” thereby ensuring strong management support. “Have management support the professionals in shaping the collaboration.”

As a key to success, participants mentioned a shared vision: “Having a shared vision in which roles, tasks, and required expertise are specified.”

The concepts that seem to form the basis for what participants consider essential to the pursuit of a common goal are as follows: *Systems Thinking, Collaborative Governance and Empowerment Theory*. Systems thinking involves understanding organizations as interconnected systems in which changes in one part affect the entire organization. This approach is crucial for integrating services across domains, such as employment, health, and social services [28].

Collaborative governance refers to the processes and structures that enable public agencies and stakeholders to engage collectively in decision-making. Doing so is vital for aligning goals and resources across different organizations [29]. Empowerment theory focuses on enabling individuals and organizations to gain control over their work and their decisions. Empowering professionals with the skills they need can lead to more effective collaboration and innovation [30].

### Facilitate and connect craftsmanship

In order to create a supportive and effective integrated approach for individuals to navigate their employment and their recovery simultaneously, participants formulated the following requirements for achieving effectiveness: “Involve professionals, managers, clients and experts with lived experience in policy-making” and “Schedule frequent case meetings in which different services review cases based on their expertise and then discuss difficulties, options, and approaches that could be used.”

By utilizing various funding sources: “Bring together different funding streams.” Services such as Individual Placement and Support (IPS) [26,27] can also be more effectively deployed.

### Observe the impact of work on patients’ health and recovery

To support individuals with mental vulnerabilities in securing employment, it is vital that caregivers are aware that being unemployed is a determinant to patients’ health, and that being employed is a critical factor that influences individuals’ health-related outcomes [31].

To operationalize an integrated approach in client-centered practice, a set of the interrelated concepts that were mentioned earlier can serve as guiding principles. Active listening serves as the foundation for understanding individuals’ lived experiences and their needs. Applying a strength-oriented and solution-focused approach helps both professionals and clients to identify small, concrete steps going forward, ensuring that goals are realistic and achievable. Emphasizing self-efficacy supports individuals in building confidence in their ability to effect change. Person-centered and individualized support ensures that services are tailored to the unique needs of each client. Empowerment and self-determination affirm individuals’ agency in this process. Upholding ethical standards, such as securing informed consent and respecting privacy and autonomy, is crucial for maintaining trust and integrity. The use of evidence-based practices strengthens the quality and accountability of interventions, while enabling a broader understanding of how various social, economic, and institutional factors interact. Finally, recognizing employment as a social determinant of health highlights the need to integrate vocational and social support into holistic care strategies.

Our study shows that promoting an integrated approach requires a drastic revision of the underlying guiding current practice that is still based on the question, How can my organization help the client? This, however, is the perspective of the organization. A next step in the development of an integrated approach would be to ask the question, How can we help the client together? But that is insufficient, because the question is still based on the perspective of care providers. The guiding principle that brings about real changes is the question, “How would you like to be helped?” This means letting go of what the care providers think should happen and making room for what is important to the client.

While Dutch policies and practice guidelines provide a framework for collaboration between mental health, social, and employment services, our findings indicate that the added value lies in how these principles are enacted in daily practice. The identified clusters highlight the importance of a collaborative mindset, shared responsibility, and a person-centred view that recognizes individuals’ strengths and recovery potential, rather than merely adhering to formal protocols.

Although the policy and service landscape has evolved since data collection in 2018, particularly through the increased use of hybrid and online collaboration, we believe the findings remain relevant. While digital communication has expanded opportunities for intersectoral collaboration, it may also reduce access to non-verbal and contextual cues that support effective communication. These cues are important for building mutual understanding, establishing trust, and facilitating shared decision-making. Consequently, although the mode of communication has changed, the fundamental challenges of collaboration identified in this study remain highly relevant and may, in some cases, be amplified in digital working environments [32].

### Strengths and limitations

There were a sufficient number of participants in the study to reflect the diversity of perspectives on promoting factors for an integrated approach for finding work for people with mental health problems. The statistical analysis yielded six clusters with the stress value within the range considered typical and acceptable for concept-mapping studies. By using focus groups, we were able to deepen our understanding of what is needed for an integrated approach.

Nevertheless, there are several limitations of the study that may limit the generalizability of the findings. The number of experts by experience and employers who responded in our concept map exercise was less than ideal. We did not contact them to determine why they did not participate. If we had done so, this might have clarified their views on the subject. To enrich our data, we added three focus groups to gain more insight into the perspectives of the other two groups.

Despite the fact that the concept map facilitated identification of factors undergirding an integrated approach and allowed multiple perspectives to be captured, it did not provide an indepth understanding of why some outcomes are of a higher priority than others. Future studies might consider conducting qualitative interviews or additional focus groups to further understand participants’ priorities.

## Conclusions

An integrated approach related to income, benefits, and care, which is aimed at securing employment for people with mental health problems requires an understanding of clients’ needs and priorities. Based on the results of our study on priorities, multiple considerations should be taken into account to improve an integrated approach. For instance, our study shows that promoting an integrated approach requires a drastic revision of the underlying guiding current practice that is still based on the question: How can my organization help the client? The guiding principle that brings about significant change is the question: How would you like to be helped?
