# Supplementary material for: How would you like to be helped? A concept mapping study of potential factors promoting integrated approaches for people with mental-health problems to find gainful employment
Source: PLoS One. 2026 Oct 6;21(10):e0357885. doi: 10.1371/journal.pone.0357885 (PMC13641371; doi:10.1371/journal.pone.0357885)
Supplement: Supplementary file 1 [file pone.0357885.s002.docx]

Here is our respons to the reviewers	6-8-2026

We sincerely thank the reviewers for their valuable time, careful evaluation, and constructive comments. We greatly appreciate their feedback, which has helped us improve the quality and clarity of our manuscript. Our detailed responses to each comment are provided in the table below.

| Reviewer #2  Abstract The abstract should include the data collection period, sampling method, key findings, and keywords. | We thank the reviewer for pointing out the missing information in the abstract. In response to this helpful suggestion, we have revised the abstract and added the requested information:  Participants were recruited between May and September 2018.  a mixed-method participatory research methodology that combines qualitative and quantitative techniques  We distinguished six clusters of potential factors that might foster an integrated approach: “Provide time and space for collaboration”, “collaborate”, “provide a smooth transition for employees”, “focus on common goals”, “facilitate and connect craftsmanship” and “pay attention to the impact of work on health and recovery”.  Key words  concept mapping study, promoting factors, integrated approaches, people with mental-health problems, gainful employment. |
| --- | --- |
| Reviewer #1  Introduction is well written. | We thank the reviewer for the positive assessment of the introduction. |
| Reviewer #2  Objective The study objective may be better incorporated into the Introduction, particularly in the final paragraph, rather than presented as a separate section. | Thank you for mentioning that the study object can be better incorporated in the final paragraph of the introduction. We removed the final paragraph and objective and added the following:  In this paper, we examine how cooperation between professionals from different domains can be further improved to develop effective integrated interventions that promote employment opportunities for people with mental health problems. Specifically, we aim to identify the factors that potentially promote an integrated approach across the domains of income, benefits, and care to support adults with mental health problems in obtaining and maintaining paid employment. Adults with mental health problems experience a wide range of diagnosable conditions that affect their thinking, emotions, mood, and behaviour, which may influence their ability to function in daily life, including work, relationships, and social interactions. By identifying these facilitating factors, this study seeks to contribute to the development of effective integrated interventions that enhance employment participation for this population. |
| Reviewer #1  Methodology is sound. Could the authors indicate how they arrived at the sample size of 106? | Thank you for asking for more clarity about how we arrived at the sample size of 106.  In the next paragraph after the added Inclusion and exclusion criteria we added:  The steering committee and task force identified and nominated eligible participants from the different stakeholder domains involved in employment support for people with mental health problems. In total, 106 participants were invited to take part in the concept mapping. |
| Reviewer #2  Methods  The Methods section should clearly describe the inclusion and exclusion criteria, and the procedures used for qualitative data analysis.  Given that recruitment took place in 2018, the authors may consider briefly discussing whether changes in the policy, service, or employment context since then could affect the current relevance or interpretation of the findings. | Thank you for pointing out to add the missing inclusion and exclusion criteria.  We added in method section;  Participants were eligible if they were professionals or experts by experience who were actively involved in daily practice and had demonstrable expertise in the domains of income, benefits and care. Participants were selected to ensure representation of the different domains involved in integrated employment support for people with mental health problems. Individuals who were not involved in daily practice or who did not have relevant expertise in the field were not eligible to participate. |
| Reviewer #2  procedures used for qualitative data analysis are not clear enough. | We thank the reviewer for this valuable comment. We have revised the Methods section to provide a clearer and more detailed description of the qualitative data analysis procedures  To make the procedure clear we added  Qualitative data analysis  The qualitative data were collected during the concept mapping sessions, in which participants generated statements in response to the focus prompt.  The focus group discussions were analyzed using an inductive qualitative content analysis. Two researchers independently reviewed the transcripts, identified key themes related to the interpretation and practical implications of the concept map, and discussed discrepancies until consensus was reached. The qualitative findings were used to enrich and contextualize the concept mapping results and are presented alongside the concept map to provide a more comprehensive understanding of the identified factors. |
| Reviewer #2  Given that recruitment took place in 2018, the authors may consider briefly discussing whether changes in the policy, service, or employment context since then could affect the current relevance or interpretation of the findings. | Thank you for this question. In the discussion we added:  Although the policy and service landscape has evolved since data collection in 2018, particularly through the increased use of hybrid and online collaboration, we believe the findings remain relevant. While digital communication has expanded opportunities for intersectoral collaboration, it may also reduce access to non-verbal and contextual cues that support effective communication. These cues are important for building mutual understanding, establishing trust, and facilitating shared decision-making. Consequently, although the mode of communication has changed, the fundamental challenges of collaboration identified in this study remain highly relevant and may, in some cases, be amplified in digital working environments. |
| Reviewer #1  Results is well presented. | Thank you for mentioning this. |
| Reviewer #2  Discussion  The authors may consider clarifying how the identified clusters contribute to the understanding of integrated approaches to employment support for people with mental health problems, and discussing how these findings relate to relevant national policies or practice guidelines on mental health, employment support, or intersectoral collaboration. | We thank the reviewer for this insightful suggestion.  We added in the discussion:  While Dutch policies and practice guidelines provide a framework for collaboration between mental health, social, and employment services, our findings indicate that the added value lies in how these principles are enacted in daily practice. The identified clusters highlight the importance of a collaborative mindset, shared responsibility, and a person-centred view that recognizes individuals' strengths and recovery potential, rather than merely adhering to formal protocols. |
| Reviewer #1  Conclusion ties in with the objectives. | We appreciate the reviewer's encouraging feedback and are glad that the conclusion effectively addresses the objectives of the study. |
| This is a comment under the acknowledgement that has to be deleted | Comment is deleted under acknowledgement and reported in the Funding Statement:  Publication of this article was supported by a grant from the Netherlands Organization for Health Research and Development (ZonMw grant number 535003006)  There was no role of the funders in study design, data collection and analysis, decision to publish, or preparation of the manuscript.  Acknowledgement is removed |
